# Elevating Restorative Dentistry: Use of the Art of Stamp Techniques in Mandibular Posterior Regions

**DOI:** 10.7759/cureus.64014

**Published:** 2024-07-07

**Authors:** Smruti S Saoji, Anuja Ikhar, Khyati Manik, Srushti Awghad, Shreeya Panchal

**Affiliations:** 1 Department of Conservative Dentistry and Endodontics, Sharad Pawar Dental College and Hospital, Datta Meghe Institute of Higher Education and Research, Wardha, IND

**Keywords:** fossa-cusp relation, bio-mimetic dentistry, occlusal morphology, occlusion, aesthetic

## Abstract

In modern dentistry, restorations that are more aesthetically pleasing have now replaced the amalgam restorations. The "stamp technique" is one of the more recently developed techniques for combining aesthetics and functionality. When caries are visible on radiographs or clinical examinations of teeth with complete marginal ridges and an ideal tooth structure, this method can be used. This novel stamp technique involves creating an index beforehand, which is essentially a miniature impression or negative copy of the occlusal topography. The advantage of the stamp technique is that it replicates the actual occlusion and its anatomy, reducing the need for additional corrections. The amount of time required for the restoration's polishing and finishing is also decreased. In comparison to manual methods, for the posterior teeth, the occlusal stamp is approachable as it is a biomimetic direct composite restoration that helps to restore hidden caries with the impaired occlusal surface. It produces better outcomes as it takes less post-restoration filling adjustments.

## Introduction

Restorations that are more aesthetically pleasing have now replaced the amalgam restorations. As amalgam has numerous downsides, which include mercury toxicity and lack of aesthetic appeal, composite restorations for posterior teeth are becoming prevalent in contemporary dentistry, going towards a time of biomimetic dentistry. "To mimic nature" is the literal meaning of the phrase "biomimetic" [1]. Composite restorations are among the most common dental surgical techniques used in routine clinical practice [2]. Since composite resins have inherent qualities, including wear characteristics, biocompatibility, and aesthetics that resemble the structure of teeth, they are frequently used for posterior permanent restorations [3]. Expertise and skill are necessary when creating an aesthetic direct composite restoration manually. The "stamp technique" is one of the more recently developed techniques for combining aesthetics and functionality [4]. The "stamp technique," put forth by a London-based dentist Dr. Waseem Riaz, is intended to facilitate the easy acquisition of precise occlusal topography in direct composite resin restorations. Even minor occlusal discrepancies following direct restorations cause discomfort for patients because the stomatognathic system's proprioceptors respond sensibly to pressure. After some time, patients make restitution by adjusting to a newly usual occlusal position, causing severe longstanding craniomandibular problems [5]. This approach is appropriate where the caries are apparent with intact marginal ridges and perfect occlusal structure during the clinical examination or routine radiographic examination of teeth [6].

This novel stamp technique involves creating an index beforehand, which is essentially a miniature impression or negative copy of the occlusal topography. It can be performed when radiographically caries are seen, but there are no obvious cavities or tooth surface loss. Before curing, this index is subsequently pressed against the last composite increment, resulting in the creation of a positive replica and the imitation of the prior condition [7]. Conventional restorative methods often involve invasive procedures and may compromise the healthy tooth structure. The stamp technique minimizes these drawbacks by utilizing a non-invasive approach that preserves more of the natural tooth. The superiority of this technique is that it replicates the actual occlusion and its anatomy, reducing the need for additional corrections. There is also a decrease in the amount of time required for the restoration's polishing and finishing [8].

## Case presentation

**Figure 1:**
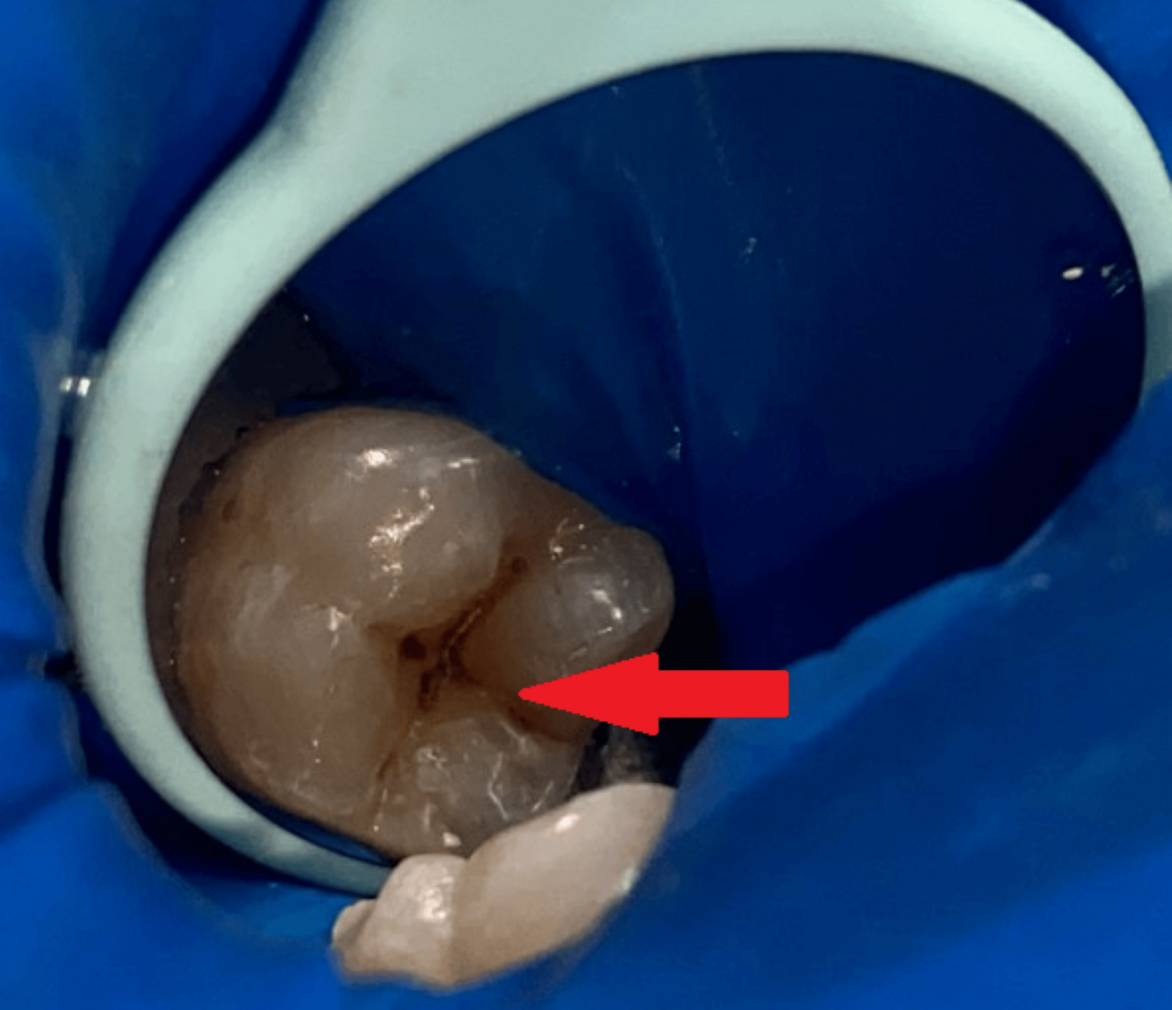
An intraoral picture shows occlusal caries in tooth number 46

A 25-year­-old female patient came to the tertiary care with a chief complaint of black-colored stains on the tooth in the mandibular right posterior region of the jaw since three months. There was no significant medical history. An oral examination revealed occlusal caries in tooth number 46 progressing to the point where the underlying dentin was visible through the enamel, manifesting as a black-colored stain (International Caries Detection and Assessment System, or ICDAS, code would likely be ICDAS 4) (Figure 1).

After performing an electric pulp test, an early response (Reading 7) was observed in the right lower first molar compared to the contralateral left lower first molar tooth (Reading 21). Following a hot gutta-percha test, where a 0.06 #15 gutta-percha (Dentsply Sirona, Charlotte, USA) was heated to 65.5°C and placed over the middle third of the mesiobuccal cusp for less than five seconds, the right lower first molar did not respond with lingering pain upon removal of the stimulus. On an intraoral periapical radiograph, radiolucency involving the enamel and dentin, without any involvement of the pulp was seen. No evidence of marginal ridge involvement was found. After a careful examination and consideration of related factors, it was determined that the tooth could be restored by utilizing the stamp technique as the stamp technique is highly prized in restorative procedures for its precision and efficiency. This method reduces patient chair time significantly, streamlining the restoration process and improving treatment efficiency overall. Moreover, it decreases the chances of errors and material waste, making it cost-effective. Its straightforward application also ensures accessibility for dental professionals, delivering superior results for a wide range of restorative requirements.

**Figure 2:**
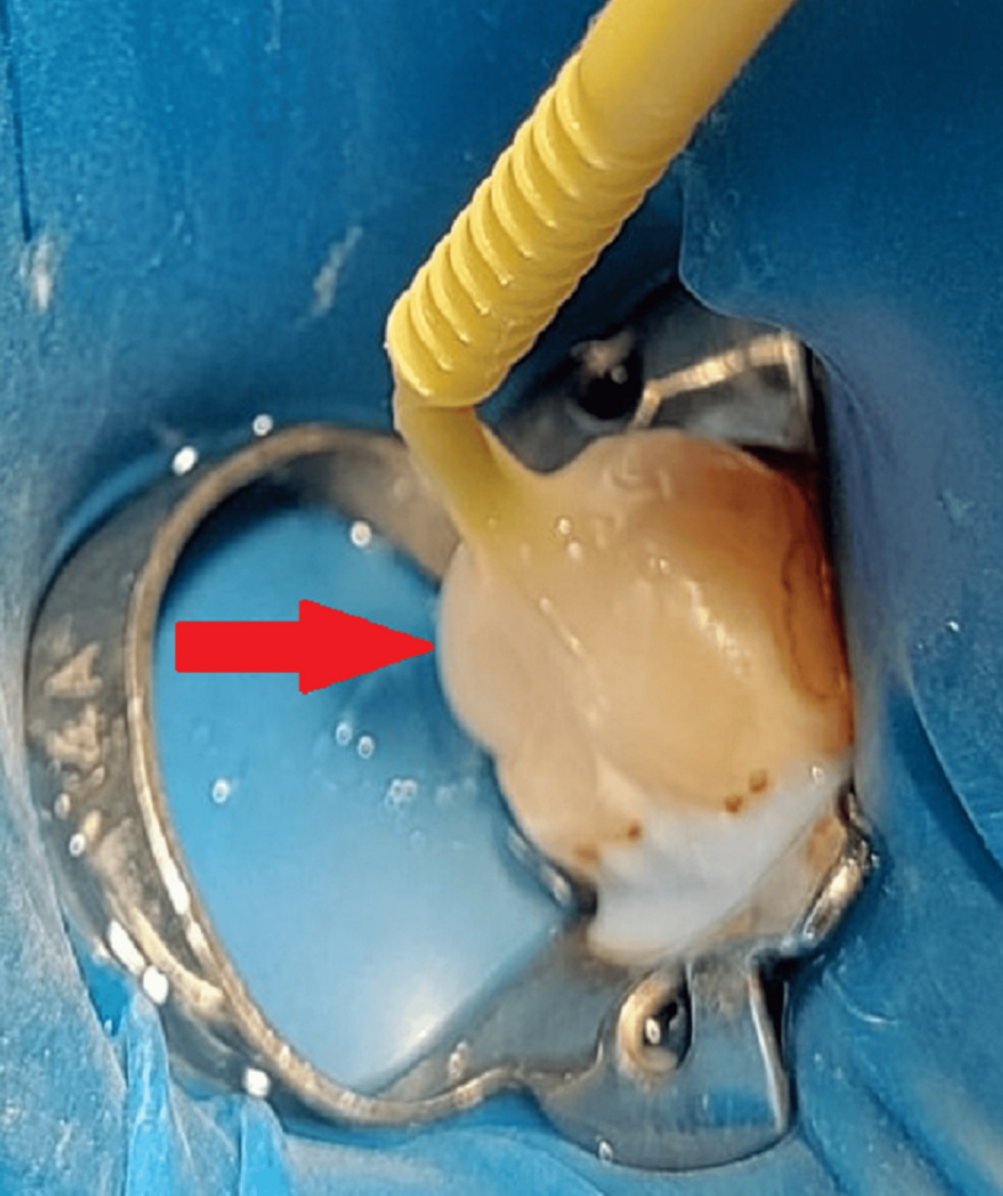
Tip of a micro-brush submerged in the composite and polymerized using light curing

A rubber dam was used to isolate the cavitated tooth. The application of the separating medium (Zartex, Zarir & Zaida Industries, Malaysia) on the occlusal surface of the tooth was done with the help of a micro-brush (Dengen Dental, USA). A stamp was produced by applying a flowable composite (Tetric N-Flow; Ivoclar Vivadent, NY, USA) to the intact occlusal surface of the tooth. To produce a stamp, the trimming of the tip of the micro-brush was done as it serves as a handle; it was then submerged in the composite and then light curing was used for polymerization (Figure 2).

**Figure 3:**
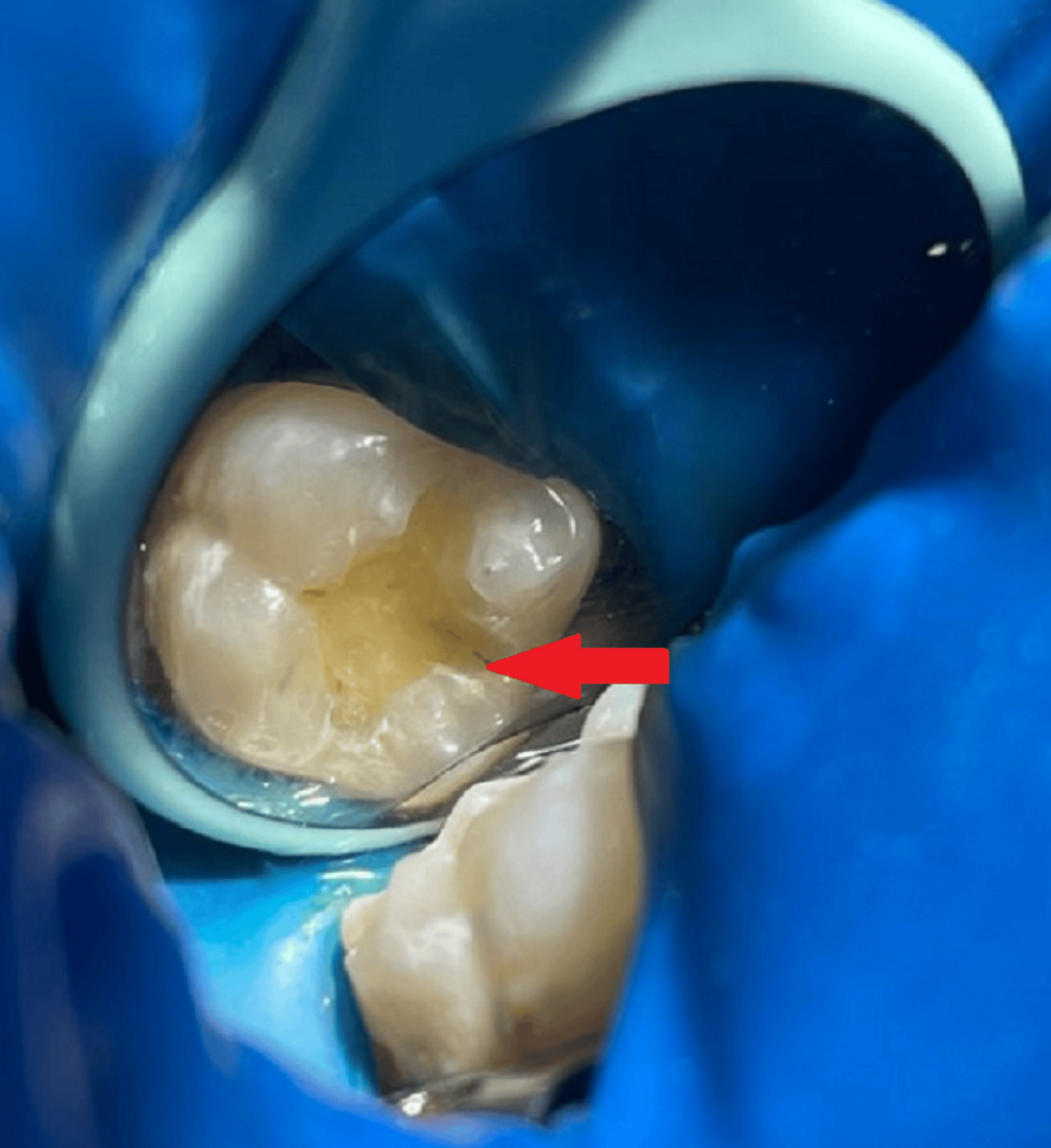
A class I cavity in tooth number 46

**Figure 4:**
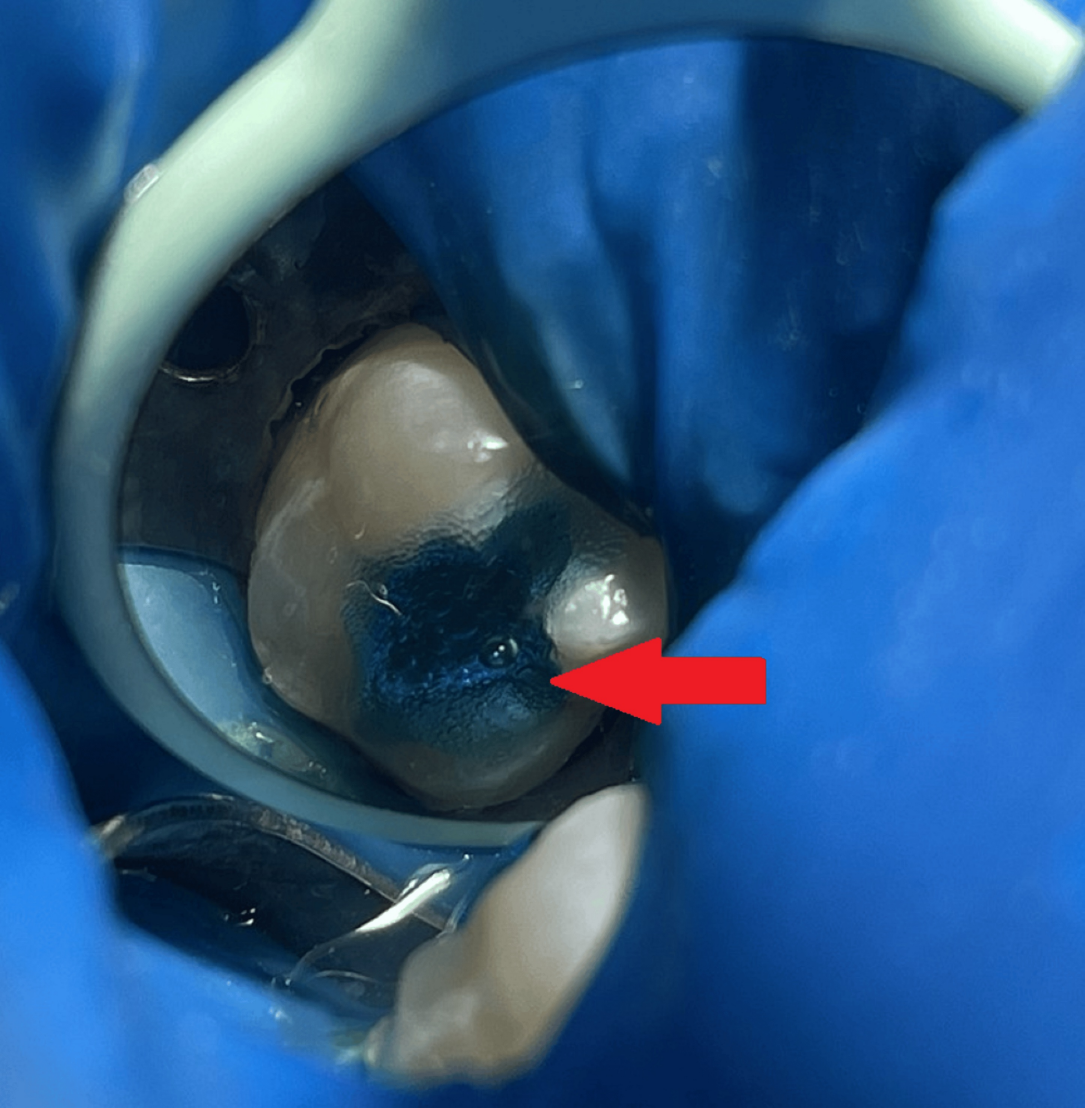
Etching with 37% orthophosphoric acid in tooth number 46

The caries excavation was done completely, and the class I cavity was achieved using an air rotor handpiece (GDC Fine Crafted Dental Pvt. Ltd., India) and a tungsten carbide bur (Figure 3). Etching was done for 30 seconds using 37% orthophosphoric acid (Tetric N-Etch; Ivoclar Vivadent) (Figure 4).

**Figure 5:**
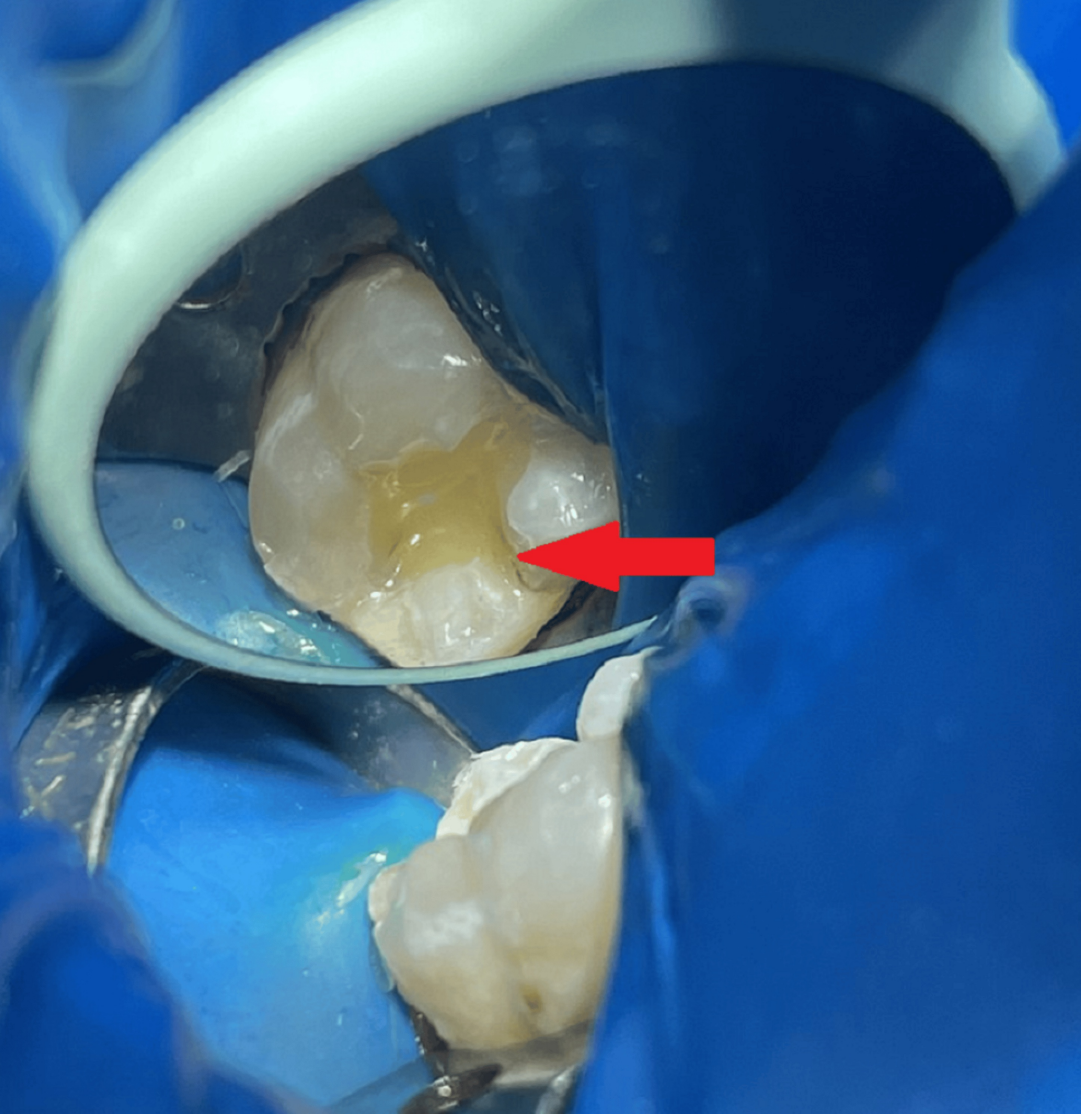
Bonding agent application in tooth number 46

It was rinsed with water for 15 to 20 seconds and then air-dried. After that, the bonding agent (Tetric N-Bond; Ivoclar Vivadent, Zurich, Switzerland) was applied using an applicator tip and then cured for 20 seconds (Figure 5).

**Figure 6:**
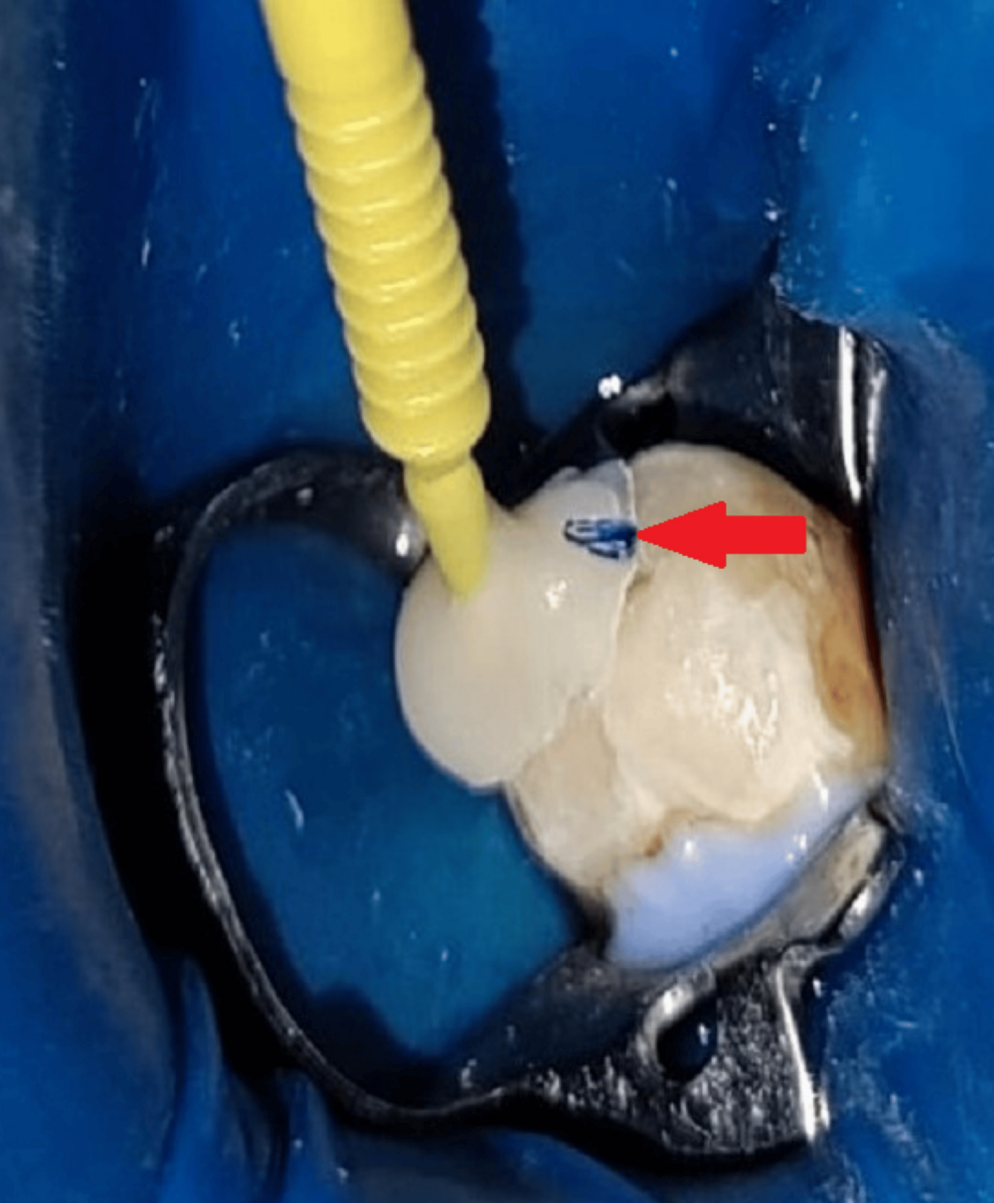
Fabricated occlusal stamp placement

The incremental approach for composite restoration was carried out up to 1 mm below the occlusal surface and light-cured for 20 seconds. A piece of Teflon sheet was placed on the tooth surface after the final increment was added and before it was cured, which serves two critical purposes. Firstly, it helps to achieve a smooth and polished surface on the restoration by preventing oxygen inhibition during the light-curing process, which could otherwise lead to a tacky layer or incomplete curing. Secondly, the Teflon sheet acts as a barrier that facilitates easy removal of excess resin and ensures that the restoration maintains its intended shape and contour, promoting optimal functional and aesthetic outcomes. The fabricated occlusal stamp was then pressed against the Teflon tape with minimal force (Figure 6).

**Figure 7:**
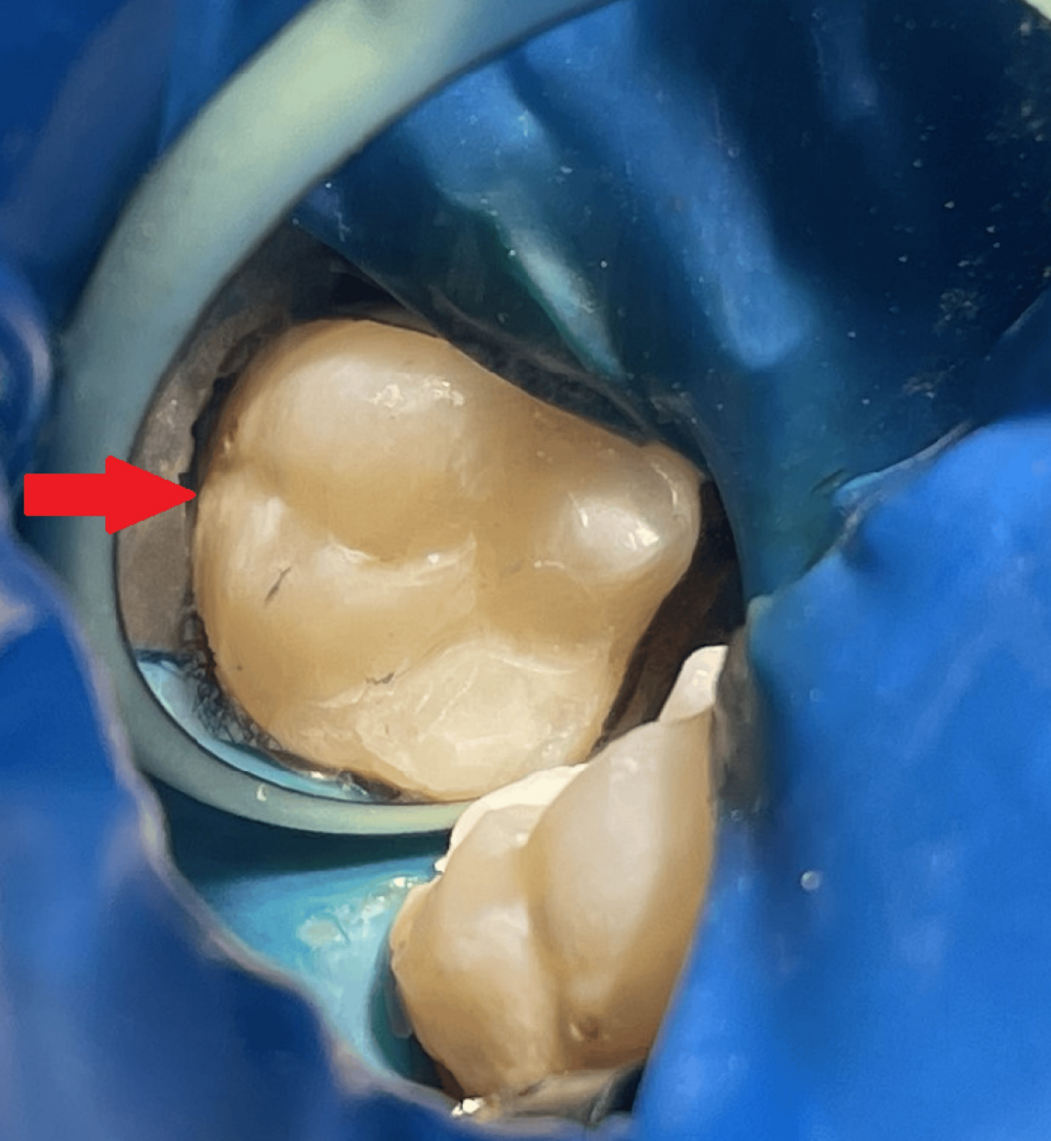
A post-operative photograph of tooth number 46

The excess material was eliminated with sharp instruments, and the resin composite was then light-cured. After removing the Teflon sheet, the final finishing and polishing were expertly carried out using a polishing paper disc (Super-Snap Mini Kit; Shofu Inc., Kyoto, Japan) (Figure 7). Using the articulating paper, the high points were checked. Post-restoration instructions were given to the patient. The patient was advised to avoid chewing on hard or sticky foods for the next 24 hours to allow the restoration to fully set. In addition, instructions were given to maintain regular oral hygiene practices, including brushing and flossing gently around the restored area to ensure long-term success and durability of the restoration.

## Discussion

To duplicate the occlusal anatomical contour of teeth, a stamp serves as an index. This approach involves creating a negative impression of the occlusal surface anatomy of a carious tooth with an intact anatomic structure. An undeniable advantage of the occlusal stamp approach is the restoration of function and aesthetics with a morphology that closely resembles the original tooth [4]. In this case, the stamp technique was chosen over manual composite restoration as a manual composite restoration technique is sensitive, lengthy, and would not result in an accurate replica of the form and occlusion. For both metallic and non-metallic restorations, different matrices are available, such as sectional matrices like tofflemire matrix bands and anatomical matrices like Palodent (Dentsply Sirona, USA) and Composi-Tight (Garrison Dental, MI, USA), which ensure precise adaptation and tight contact between the restoration and adjacent teeth for excellent clinical outcomes, primarily making it possible to achieve the proximal surfaces, contour, and contact [9]. If these matrices are not used to restore the tooth, it may fail to achieve accurate occlusion [10]. The occlusal carving is left up to the expertise and dexterity of the free hand. As the process is technique-sensitive, there is a risk of over-/underfilling of the restoration, which would lead to occlusal discrepancies that can cause trauma from occlusion [10]. The stamp technique was used in this case as the tooth structure was intact, and the occlusal caries were seen [11]. According to Tambake et al., the stamp technique takes less time [11]. The usage of materials is minimized, there is less chair time during finishing and polishing, and the anatomical morphology is closely achieved. Similarly, in this technique, there is no requirement for any special instrument to obtain the anatomical morphology of the tooth and this procedure is very simple and easy to follow [10].

The stamp technique offers a notable advantage in minimizing the total time for which the material remains under pressure, resulting in faster completion of finishing procedures post-recovery. The occlusal stamp approach for posterior teeth produces better results and requires fewer post-restoration filling corrections to replace hidden cavities with entire occlusal surfaces when compared to manual procedures [12]. This method is beneficial as it enhances the strength of the enduring tooth structure and the attainability of repair. Alshehadat et al. mentioned that to get a good fossa-cusp relation with the antagonist dentition, minimal time is required in the stamp technique; for busy practitioners, this is a blessing and enhances their credibility with patients [6]. Moreover, in the finished restoration, the level of porosity is significantly lower. The stamp technique addresses issues with composite polymerization by utilizing a Teflon sheet to prevent oxygen inhibition, ensuring complete curing without a sticky layer. This method promotes a smoother restoration surface and reduces the risk of material shrinkage or incomplete polymerization, enhancing durability and aesthetic results [13]. These attributes are suggested as important indicators influencing the enduring success of composite dental restorations [14]. The flowable composite is used to prepare the stamp as it penetrates every irregularity and improves cavity adaptation. It also has excellent handling characteristics and has high strength, which reduce the chance of stamp breakage [15].

However, it is important to consider the downsides, such as the susceptibility to stamp breakage and the cost associated with the flowable composite. The drawback of this technique is that to be executed successfully, it requires expertise and clinical judgment. It is also contraindicated in grossly carious teeth. Additionally, the process demands a costly flowable composite and micro-brushes. It is possible to get around this drawback by using transparent acrylic resin or expired flowable composites [16].

## Conclusions

To conclude, in comparison to manual methods, the stamp technique is a revolutionary biomimetic direct composite restoration procedure for posterior teeth that helps restore hidden cavities with intact occlusal surfaces in a shorter time and with fewer adjustments after restoration.
